# Using convolutional neural networks to detect GNSS multipath

**DOI:** 10.3389/frobt.2023.1106439

**Published:** 2023-05-11

**Authors:** Anthony Guillard, Paul Thevenon, Carl Milner

**Affiliations:** ^1^ Ecole Nationale de l’Aviation Civile (ENAC), SIGNAV, Toulouse, France; ^2^ 3D Aerospace, Toulouse, France

**Keywords:** Global Navigation Satellite System, convolutional neural network, multipath, machine learning, correlator, DLL

## Abstract

Global Navigation Satellite System (GNSS) multipath has always been extensively researched as it is one of the hardest error sources to predict and model. External sensors are often used to remove or detect it, which transforms the process into a cumbersome data set-up. Thus, we decided to only use GNSS correlator outputs to detect a large-amplitude multipath, on Galileo E1-B and GPS L1 C/A, using a convolutional neural network (CNN). This network was trained using 101 correlator outputs being used as a theoretical classifier. To take advantage of the strengths of convolutional neural networks for image detection, images representing the correlator output values as a function of delay and time were generated. The presented model has an F score of 94.7% on Galileo E1-B and 91.6% on GPS L1 C/A. To reduce the computational load, the number of correlator outputs and correlator sampling frequency was then decreased by a factor of 4, and the convolutional neural network still has an F score of 91.8% on Galileo E1-B and 90.5% on GPS L1 C/A.

## 1 Introduction

Global Navigation Satellite Systems (GNSSs) are at the heart of the positioning applications. These applications are progressively catered toward highly accurate solutions. However, in urban and suburban environments, the challenge for an accurate positioning solution persists. Indeed, cycle slips on carrier phase measurements (Takasu Tomoji, 2008) and large multipath errors on pseudoranges frequently occur. This paper aims at mitigating the effect of large biases introduced to the pseudoranges by multipath.

Multipath can be divided into two categories (Lau, 2021):

• Only Non-Line-of-Sight (NLOS) signals arriving to the antenna.
• A Line-of-Sight (LOS) signal with one or multiple reflections of the LOS signal.

In the existing literature, most algorithms focus on the detection of one category at a time with a heavy focus on NLOS detection with a wide array of techniques. Hsu (2017) tried to detect NLOS satellites with a support vector machine (SVM). Jiang et al. (2021) aimed at modeling the code discriminator with Gaussian fitting to detect NLOS. Sanromà Sánchez et al. (2016) used fisheye images and the carrier-to-noise density ratio (*C*/*N*_0_). This is due to the fact that NLOS signals and multipath interference often affect the positioning solution very differently (Groves et al., 2013). Some account for LOS and NLOS reflections in the same algorithm such as the study by Matera et al. (2019), where the multipath is isolated from reference base stations data fused with fisheye images, or the study by Suzuki et al. (2020), where a rotating antenna is used to mitigate both categories of multipath errors.

Lately, machine learning (ML) has been applied to GNSS data processing especially for multipath detection/mitigation. For example Munin et al. (2020) and Blais et al. (2022) fed synthetic correlator outputs to a convolutional neural network (CNN) to detect multipath. Since they generated controlled multipath and knew when multipath affected the correlators, their method lacked a classification process that made the method applicable to real signals. Furthermore, images containing the correlator outputs fed to the CNN were generated from every integration epoch of the tracking process. However, in real-life scenarios, due to the tracking filters and multipath, correlator outputs are correlated in time. Suzuki and Amano (2021) also used correlator outputs to detect NLOS *via* neural networks and SVMs by classifying NLOS measurements using a fisheye camera-based algorithm. They, too, used correlator outputs at every integration epoch, leading to correlation between the machine learning inputs. Quan et al. (2018) also used CNNs for multipath detection/exclusion but used the signal-to-noise ratio and pseudoranges as inputs to the network. Xu et al. (2020) and Hsu (2017) used SVMs to detect NLOS using shadow matching and GNSS measurements (pseudoranges and Doppler shifts), respectively.

In this article, the goal is to detect LOS reflections and then exclude them in real signals (Galileo E1-B and GPS L1 C/A) using GNSS data only. LOS reflections happen very often in challenging environments and excluding all affected measurements would lead to a low positioning availability, and not all LOS reflections severely degrade the positioning solution (Prochniewicz and Grzymala, 2021). The definition of multipath depends on the targeted positioning accuracy. The definition used in this paper will be defined in section 2.2 and will also take into account the type of materials encountered in deep urban conditions. Multipath rays depend on the environment; they cannot be predicted unless 3D maps are built and/or ray-tracing is used (Bétaille et al., 2013; Peyret et al., 2014). The detection of NLOS multipath is not targeted because the classification criterion proposed herein does not allow for precise NLOS detection. This detection process can be obtained by other methods such as the ones presented in Sanromà Sánchez et al. (2016), Suzuki and Amano (2021), or Matera et al. (2019). As no external information regarding the environment is used in this paper, using CNNs can be very efficient in finding features that are hard to model (Liu, 2018). The convolutional neural network model was used as a binary classifier:

• 0: No multipath present
• 1: Presence of multipath

The CNN inputs are obtained due to correlator output values as a function of their delay (with respect to the prompt correlator) and as a function of time yielding a 2D image. As CNNs are particularly efficient in image analysis (Jogin et al., 2018), these images are fed to the CNN proposed herein.

For any supervised neural network to be trained, it needs pre-labeled data. Without this preliminary classification, the CNN cannot operate successfully. The labeling approach taken in this paper is to use multiple correlator outputs from the tracking stage to compute the discriminator function. The tracking error is then used to determine whether the measurement is affected by multipath or not. This method is further detailed in section 2.1. The computational gain that CNNs bring with respect to computing the tracking bias is also investigated.

## 2 Materials and methods

### 2.1 Multipath error on the tracking process

For a single GNSS satellite, the arriving signal has to be acquired and then tracked so that its data bits can be demodulated. After tracking without multipath, the expression of the in- and quadrature-phase (I and Q) correlator for the prompt, early, and late correlator outputs are as follows:

$$\begin{array}{cc} I_{P}\left(k\right) & =\frac{A\left(k\right)}{2}d\left(k\right)R_{c}\left(\varepsilon_{\tau \left(k\right)}\right)sinc\left(\pi \varepsilon_{f\left(k\right)}T_{\mathrm{coh}}\right)\\ & \;\times cos\left(\varepsilon_{\theta \left(k\right)}\right)+\varepsilon_{noise,I_{P}}\left(k\right)\\ I_{E}\left(k\right) & =\frac{A\left(k\right)}{2}d\left(k\right)R_{c}\left(\varepsilon_{\tau \left(k\right)}+\frac{E_{L}}{2}\right)sinc\left(\pi \varepsilon_{f\left(k\right)}T_{\mathrm{coh}}\right)\\ & \;\times \cos\left(\varepsilon_{\theta \left(k\right)}\right)+\varepsilon_{noise,I_{E}}\left(k\right)\\ I_{L}\left(k\right) & =\frac{A\left(k\right)}{2}d\left(k\right)R_{c}\left(\varepsilon_{\tau \left(k\right)}-\frac{E_{L}}{2}\right)sinc\left(\pi \varepsilon_{f\left(k\right)}T_{\mathrm{coh}}\right)\\ & \;\times \cos\left(\varepsilon_{\theta \left(k\right)}\right)+\varepsilon_{noise,I_{L}}\left(k\right), \end{array}$$ (1)

$$\begin{array}{cc} Q_{P}\left(k\right) & =\frac{A\left(k\right)}{2}d\left(k\right)R_{c}\left(\varepsilon_{\tau \left(k\right)}\right)sinc\left(\pi \varepsilon_{f\left(k\right)}T_{\mathrm{coh}}\right)\\ & \;\times \sin\left(\varepsilon_{\theta \left(k\right)}\right)+\varepsilon_{Noise,Q_{P}}\left(k\right)\\ Q_{E}\left(k\right) & =\frac{A\left(k\right)}{2}d\left(k\right)R_{c}\left(\varepsilon_{\tau \left(k\right)}+\frac{E_{L}}{2}\right)sinc\left(\pi \varepsilon_{f\left(k\right)}T_{\mathrm{coh}}\right)\\ & \;\times \sin\left(\varepsilon_{\theta \left(k\right)}\right)+\varepsilon_{Noise,Q_{E}}\left(k\right)\\ Q_{L}\left(k\right) & =\frac{A\left(k\right)}{2}d\left(k\right)R_{c}\left(\varepsilon_{\tau \left(k\right)}-\frac{E_{L}}{2}\right)sinc\left(\pi \varepsilon_{f\left(k\right)}T_{\mathrm{coh}}\right)\\ & \;\times \sin\left(\varepsilon_{\theta \left(k\right)}\right)+\varepsilon_{Noise,Q_{L}}\left(k\right), \end{array}$$ (2)

where

• *I*_*X*_ is the in-phase correlator (prompt *P*, early *E*, and late *L*)
• *Q*_*X*_ is the quadrature phase correlator (prompt *P*, early *E*, and late *L*)
• *A* is the amplitude of the incoming signal
• *R*_*c*_ is the autocorrelation function of the pseudorandom noise (PRN) code
• *E*_*L*_ is the early–late spacing in chips
• *k* is the discrete integration time interval
• *ɛ*_*θ*(*k*)_ is the difference between the estimated phase and the phase of the incoming signal
• *ɛ*_*f*(*k*)_ is the difference between the estimated frequency and the frequency of the incoming signal
• *ɛ*_*τ*(*k*)_ is the difference between the estimated delay and the delay of the incoming signal
• *T*_*coh*_ is the coherent integration time
• $\varepsilon_{Noise,I_{X}}\left(k\right)$ is the noise for the correlator *I*_*X*_
• $\varepsilon_{Noise,Q_{X}}\left(k\right)$ is the noise for the correlator *Q*_*X*_

When tracking a GNSS signal, the delay-locked loop (DLL) and phase-locked loop (PLL) use discriminators to retrieve the delay and phase error, respectively. The DLL tries to estimate the travel time of the incoming signal by matching a locally generated code with a known time delay, if the delay is within one PRN code length. When the signal is emitted from the satellite, the satellite clock delay and other hardware delays are added onto the signal travel time. The atmospheric delays induced by different media are also accounted for in the travel time. When estimating the travel time of the incoming signal with the discriminator, the atmospheric errors and the satellite clock bias shift the value of the travel time but do not distort the autocorrelation function (ACF) of the PRN code.

To retrieve the delay error, the DLL uses at least three correlators (early, prompt, and late). The delay error is used to refine the frequency of the locally generated replica with the incoming signal. If the locally generated code replica is aligned with the incoming PRN sequence, then the early and late correlator values are equal in an error-free scenario. The difference in chips between the early and late correlator positions is called the early–late (EL) spacing. This parameter has a quintessential role for the DLL as a large EL spacing will lead to a better tracking of the dynamics, while a smaller one will reduce the effect of multipath (Van Dierendonck et al., 1992).

In this study, only non-coherent discriminators are used as they do not make any assumption on the state of the PLL—on the phase error. The discriminator function is given for the EL power, a non-coherent discriminator. In the absence of noise and multipath, its function is given as follows:

$$D_{\mathrm{EMLP}}\left(\tau \right)=\frac{I_{E}^{2}+Q_{E}^{2}-I_{L}^{2}-Q_{L}^{2}}{2},$$ (3)

$$\begin{array}{cc} D_{\mathrm{EMLP}}\left(\tau \right) & =\frac{A{\left(k\right)}^{2}}{4}sinc{\left(\pi \varepsilon_{f\left(k\right)}T_{\mathrm{coh}}\right)}^{2}\\ & \;\times \left({\left|R_{c}\left(\tau +\frac{E_{L}}{2}\right)\right|}^{2}-{\left|R_{c}\left(\tau -\frac{E_{L}}{2}\right)\right|}^{2}\right), \end{array}$$ (4)

where

• *E*_*L*_ is the early–late spacing in chips

**FIGURE 1:**
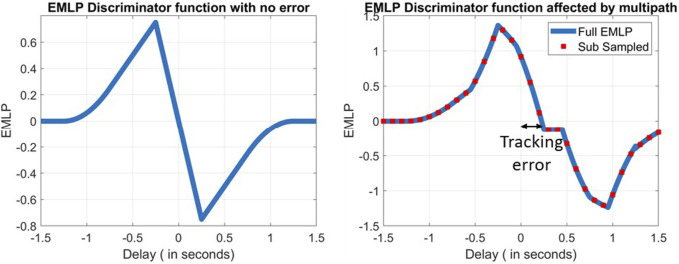
Early–late power discriminator function in error-free conditions (left) and affected by some multipath (right).

The value of *τ* for *D*_*EMLP*_(*τ*) = 0 yields the extra delay caused by the errors affecting the DLL. To find this delay, the zero crossing of the discriminator must be found. The slope of the EMLP discriminator must be negative for it to be a stable lock point. As the autocorrelation function of the PRN code is an even function (∀x∈R s.t f(x)=y & f(−x)=y) and considering that the signal is unaffected by multipath and ignoring noise, the only possible value of *τ* is zero. Therefore, the discriminator will have a zero crossing where the tracking delay is equal to 0 as shown in Figure 1 (left).

In a realistic scenario, tracking errors will always be present in GNSS receivers. Noise is not the sole cause for errors being propagated from the tracking stage to the pseudorange but also multipath. As conveyed by Bellad and Petovello (2013), multipath can distort the ACF. This distortion depends on the number of multipaths, their phase, their amplitude, and their delay. When the ACF is distorted, this shifts the zero crossing of the discriminator function as well. This is illustrated in Figure 1.

Indeed, when adding multipath, (1) and (2) become:

$$\begin{array}{cc} I_{P}\left(k\right) & =\overset{N}{\underset{i=1}{\sum }}\frac{A_{i}\left(k\right)}{2}d\left(k-\tau_{i}\right)R_{c}\left(\varepsilon_{\tau_{i}\left(k\right)}\right)\\ & \;\times sinc\left(\pi \varepsilon_{f_{i}\left(k\right)}T_{\mathrm{coh}}\right)\cos\left(\varepsilon_{\theta_{i}\left(k\right)}\right)+\varepsilon_{noise,I_{P}}\left(k\right)\\ I_{E}\left(k\right) & =\overset{N}{\underset{i=1}{\sum }}\frac{A_{i}\left(k\right)}{2}d\left(k-\tau_{i}\right)R_{c}\left(\varepsilon_{\tau_{i}\left(k\right)}+\frac{E_{L}}{2}\right)\\ & \;\times sinc\left(\pi \varepsilon_{f_{i}\left(k\right)}T_{\mathrm{coh}}\right)\cos\left(\varepsilon_{\theta_{i}\left(k\right)}\right)+\varepsilon_{noise,I_{E}}\left(k\right)\\ I_{L}\left(k\right) & =\overset{N}{\underset{i=1}{\sum }}\frac{A_{i}\left(k\right)}{2}d\left(k-\tau_{i}\right)R_{c}\left(\varepsilon_{\tau_{i}\left(k\right)}-\frac{E_{L}}{2}\right)\\ & \;\times sinc\left(\pi \varepsilon_{f_{i}\left(k\right)}T_{\mathrm{coh}}\right)\cos\left(\varepsilon_{\theta_{i}\left(k\right)}\right)+\varepsilon_{noise,I_{L}}\left(k\right), \end{array}$$ (5)

$$\begin{array}{cc} Q_{P}\left(k\right) & =\overset{N}{\underset{i=1}{\sum }}\frac{A_{i}\left(k\right)}{2}d\left(k-\tau_{i}\right)R_{c}\left(\varepsilon_{\tau_{i}\left(k\right)}\right)\\ & \;\times sinc\left(\pi \varepsilon_{f_{i}\left(k\right)}T_{\mathrm{coh}}\right)\sin\left(\varepsilon_{\theta_{i}\left(k\right)}\right)+\varepsilon_{noise,Q_{P}}\left(k\right)\\ Q_{E}\left(k\right) & =\overset{N}{\underset{i=1}{\sum }}\frac{A_{i}\left(k\right)}{2}d\left(k-\tau_{i}\right)R_{c}\left(\varepsilon_{\tau_{i}\left(k\right)}+\frac{E_{L}}{2}\right)\\ & \;\times sinc\left(\pi \varepsilon_{f_{i}\left(k\right)}T_{\mathrm{coh}}\right)\sin\left(\varepsilon_{\theta_{i}\left(k\right)}\right)+\varepsilon_{noise,Q_{E}}\left(k\right)\\ Q_{L}\left(k\right) & =\overset{N}{\underset{i=1}{\sum }}\frac{A_{i}\left(k\right)}{2}d\left(k-\tau_{i}\right)R_{c}\left(\varepsilon_{\tau_{i}\left(k\right)}-\frac{E_{L}}{2}\right)\\ & \;\times sinc\left(\pi \varepsilon_{f_{i}\left(k\right)}T_{\mathrm{coh}}\right)\sin\left(\varepsilon_{\theta_{i}\left(k\right)}\right)+\varepsilon_{noise,Q_{L}}\left(k\right), \end{array}$$ (6)

where

• *i* is the index of the signal received (direct or reflected)
• *N* is the number of multipath reflections
• *τ*_*i*_ is the delay error introduced by multipath i in seconds

Due to the several multipath delays, the zero crossing for the discriminator function is not at *τ* = 0 anymore. This is illustrated in Figure 1 (right).

By reconstructing the ACF of each tracking epoch with multiple correlators, the discriminator function can be obtained with great accuracy. However, if the discriminator function is subsampled, the tracking error has to be linearly interpolated at the zero crossing. The further the correlators are spaced from one another, the less accurate the interpolation is. This phenomenon is depicted in red in Figure 1 (right).

When the number of correlators is insufficient or does not cover enough of the ACF, the delay may not be computed due to the lack of zero crossing. In this study, when this occurs (only with low correlator resolutions), the delay is assumed to be zero to not reset the low pass filter.

The discriminator function can have several zero crossings. The zero crossing that is the closest to the prompt index and with a negative slope is chosen as the stable lock point from which to compute the tracking bias—since the EMLP discriminator is used. On the presented dataset, when this occurs, the second zero crossing is much further in terms of delay than the one chosen as the stable point with respect to the prompt correlator.

When the computed tracking delay in seconds is multiplied by the speed of light, the tracking bias is obtained in (7). Once this bias is passed through the low-pass filter of the tracking process, this filtered bias is propagated onto the pseudorange measurement as follows:

$$\exists t_{0}\in \left[-T_{\mathrm{obs}},T_{\mathrm{obs}}\right]\text{ such that }D\left(t_{0}\right)=0,$$

$${\mathrm{Tr}}_{b}=ct_{0},$$ (7)

$$\rho_{b}=LPF\left({\mathrm{Tr}}_{b}\right),$$

where

• *T*_*obs*_ is the maximum delay for which the discriminator is observed
• *c* is the speed of light in m/s
• Tr_*b*_ is the tracking bias in meters
• *LPF* is the low-pass filter of the DLL modelled by a Butterworth filter of order 2 with a bandwidth of *B*_*DLL*_
• *ρ*_*b*_ is the pseudorange bias induced from the tracking bias in meters

### 2.2 Theoretical classification

In this paper, up to 101 correlators were used to reconstruct the ACF of the PRN code. Only the non-coherent discriminator will be studied because this is the most robust type of discriminator since it does not assume any PLL lock.

In a DLL where only additive white Gaussian noise affects the signal, the propagated noise can be estimated as zero mean Gaussian noise (Legrand et al., 2000). For the early–late power discriminator, which is non-coherent, the variance of the noise can be expressed as follows:

$$\begin{array}{c} \sigma_{\mathrm{Noise}}^{2}=T_{c}^{2}\frac{B_{\mathrm{DLL}}E_{L}}{2C/N_{0}}\left[1+\frac{2}{\left(2-E_{L}\right)T_{\mathrm{coh}}C/N_{0}}\right], \end{array}$$ (8)

where

• *T*_*c*_ is the chip length in meters
• *B*_*DLL*_ is the equivalent loop filter bandwidth of the DLL in Hz
• *E*_*L*_ is the EL spacing in chips
• *C*/*N*_0_ is the carrier-to-noise-density ratio in Hz
• *T*_*coh*_ is the coherent integration time in seconds
• $\sigma_{\mathrm{Noise}}^{2}$ is the variance of the DLL noise in meters ^2^

Since the DLL noise is assumed to follow a Gaussian law with a known distribution $\left(\epsilon_{\mathrm{Noise}}∼N\left(0,\sigma_{\mathrm{Noise}}^{2}\right)\right)$ , it implies that:

$$\epsilon_{\mathrm{noise}}\leq 3\times \sigma_{\mathrm{Noise}}\text{ for }99.7\text{\% of the time}.$$ (9)

The transient errors are ignored, so the tracking bias is assumed to be only caused by multipath and noise, which can then be expressed as follows (Braasch, 2017):

$$\rho_{b}\approx \epsilon_{MP}+\epsilon_{\mathrm{Noise}}.$$ (10)

Hence, any bias exceeding 3 × *σ*_*Noise*_ can be considered to be caused by multipath. However, labeling every error exceeding this threshold would lead to a lot of measurements being classified as affected by multipath, especially in urban and suburban environments. If excluded, this would not only leave the most accurate measurements for positioning but would surely leave very few measurements for the positioning solution. In GNSS, a minimum of four satellites, when one constellation is used, is needed to compute a positioning solution (plus one extra satellite per additional constellation). If too many satellites are excluded due to being labeled as affected by multipath, the following may occur:

• Not enough satellites to compute a position
• An increase in the dilution of precision values

On the other hand, if faulty measurements are not excluded from the positioning, then the positioning solution cannot be trusted.

To establish a new threshold, the idea was to find a value that would be small enough to not cause a large bias in the pseudorange but also large enough to not cause availability issues due to frequent multipath labeling. As the largest availability *vs.* multipath challenge occurs in degraded environments, the multipath sources can be identified. Indeed, multipath will often come from buildings, roads, or other vehicles. When looking at attenuation factors of common surfaces at a normal incidence, a threshold can be established based on materials that yield a tolerable multipath error. The values of these materials, for the L1 frequency, are given as Table 1 follows and available in the study by Braasch (2017). By retrieving the attenuation factor from the table, the maximum amplitude of the multipath coming from this material can be known. Therefore, the multipath error envelope (MEE) can be computed for a given material. The MEE indicates how large the tracking bias can be for a given relative multipath amplitude factor and early–late spacing as a function of the multipath delay. When plotting the MEE, two curves are represented. The upper curve represents the error when the multipath is in phase, and the lower curve depicts the error when the multipath is out of phase. All multipath errors for the given parameters (relative amplitude factor and early–late spacing) are contained within the two bounds. Hence, the maximum induced multipath bias by a given material is given as follows:

$$b_{\mathrm{Material}}=\max\left(\left|M_{EE}\left(t_{MP};E_{L},\alpha_{\mathrm{Material}}\right)\right|\right),$$ (11)

where

• *M*_*EE*_ is the multipath error envelope value for a given early–late spacing and relative multipath amplitude factor
• *t*_*MP*_ is the multipath delay in chips
• *b*_*Material*_ is the maximum multipath error caused by reflection from a given material
• *α*_*Material*_ is the relative multipath amplitude factor of a given material

**TABLE 1:** Reflection and attenuation factors for common urban surfaces at normal incidence on L1 frequency.

Surface	Attenuation factor (dB)
Asphalt	−18.3
Brick	−9.24
Concrete	−7.87
Glass	−7.51
Tinted glass	−0.446

**FIGURE 2:**
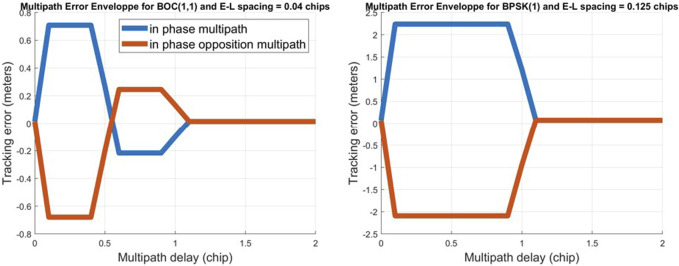
Multipath error envelope for a brick-induced multipath for an EL spacing of 0.04 chips on Galileo E1-B (left) and for an EL spacing of 0.125 chips on GPS L1 C/A (right).

For example, the MEE of brick is given in Figure 2 for an EL spacing of 0.04 chips for Galileo E1-B (left) and the MEE of brick given in Figure 2 (right) is for an EL spacing of 0.125 chips for GPS L1 C/A.

As assumed in the study by Zhao et al. (2013), the most commonly encountered materials in challenging environments will be asphalt, glass, tinted glass, brick, and concrete. Brick is assumed to not be the most common building material. Since it has a high attenuation coefficient, it was decided that any multipath that has lower amplitude than the ones generated from brick would be tolerated. However, this method still functions if the chosen material is different as its selection depends on the desired multipath detection sensitivity. Relating to Figure 2 (left), this means that any multipath, for a DLL with an EL spacing of 0.04 chips, that causes a bias smaller than 0.708 m would be tolerated.

The early–late spacing used on GPS L1 C/A was higher than that for Galileo E1-B as a low EL spacing on L1 C/A led to a prolonged convergence of the tracking loops of the receiver. This change also induces an increase in the bias of (12), meaning that the threshold for multipath classification is higher on GPS L1 C/A than Galileo E1-B (*b*_*Material*_ = 2.23 m). As shown by Prochniewicz and Grzymala (2021), GPS L1 C/A is more affected by multipath than Galileo E1-B, so this rise in threshold for GPS L1 C/A makes sense to keep a high availability.

Thus, the threshold for multipath can be written as follows:

$$T=3\times \sigma_{\mathrm{Noise}}+b_{\mathrm{Brick}},$$ (12)

where

• *b*_*Brick*_ is the maximum multipath error caused by a reflection from brick

This gives the following classification:

$$\text{Decision}=\left\{\begin{array}{c} |\rho_{b}|\geq T\to \text{Multipath}\;\\ |\rho_{b}|<T\to \text{Not Multipath}.\; \end{array}\right.$$ (13)

As shown in the study by Vergara et al. (2009), the DLL noise increases due to the presence of multipath. However, the increase in noise is assumed to be accounted for by the addition of *b*_*brick*_.

The decision given in (13) will be the method used to classify multipath and train the convolutional neural network presented in section 2.4.

### 2.3 Signal deformation

**FIGURE 3:**
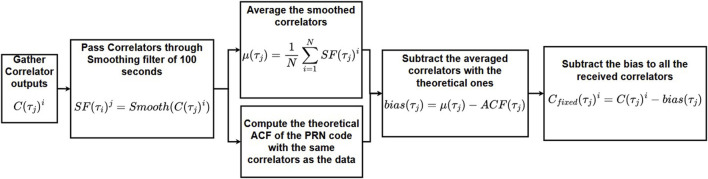
PRN deformation correction algorithm.

When first plotting the tracking biases and its filtered version, it was noticed that they were not centered around zero. These biases were slightly offset by a different value depending on the satellite number and constellation. Because of this, setting a threshold would not be possible according to the decision given in (14). This tracking bias is caused by the digital and/or analog hardware distortions (Song et al., 2020). Pagot et al. (2015) used the S-crossing of the discriminator among other techniques to quantify the bias. Pagot et al. (2017) used in-phase correlators to detect the deformation of the signal. In the study by Fan and Mats Brenner (2006), their solution estimated the satellites and receiver hardware biases by estimating the bias caused on each correlator. However, it required several identical receivers to do so, which was not possible in this study. Thus, the proposed solution here is a modified version of that in the study by Fan and Mats Brenner (2006) and estimates the satellite and receiver biases as one bias altogether. This method assumes that the hardware and receiver biases are constant over the duration of the dataset. They are also assumed to be the same over other data collected, even though they will vary according to temperature and the incidence angle. These supposedly small variations are assumed to be accounted for in the standard deviation of the noise, so in the threshold of (13). The method is presented in Figure 3.

### 2.4 Convolutional neural network training

#### 2.4.1 CNN theory

The accuracy of the tracking bias in GNSS receivers is limited by the sampling frequency in the correlation domain of the signal. The receiver used in this study, presented in section 2.5, can have a correlation domain sampling frequency of 200 MHz at most. A total of 101 correlators at 200 MHz were used to compute the most accurate tracking bias possible with the equipment used. Furthermore, computing an accurate tracking bias in GNSS receivers is time consuming due to the large number of correlators needed. Therefore, this method is best reserved for theoretical classification, but a more lightweight solution is needed to be more applicable to GNSS receivers. Training a CNN can be cumbersome, so it only has to be done once. When trained, the model can then be used in receivers as the prediction is fast. CNN images can be constructed with fewer correlators than used for the theoretical classification presented in section 2.2. Evidently, reducing the number of correlators affects the accuracy of the model as discussed in section 3.Despite having a large freedom of implementation for CNNs, all models share some properties and layers which are listed as follows:

1) Convolutional layer
2) Activation function
3) Pooling layer
4) Output layer
5) Loss function

As the name indicates, convolutional neural networks rely on the convolution operation. The input, for example, an image, is a 2D matrix (or 3D if in color). This input is then convolved with multiple filters of the same size but of different values. Filters can be thought of as feature-detection matrices. In computer vision, kernels can be used to detect edges or sharpen the images (Lampert, 2009). CNNs are based on the same principle but keep updating the values of their filters to detect the most prominent features of the input images. The number of filters used in a convolutional layer will determine the number of 2D outputs. These 2D outputs are called feature maps since each one was passed through a different filter, thus detecting different features. The mathematical expression of a convolutional layer is given as follows (Carneiro et al., 2017):

$$A_{o}^{m}=\overset{N}{\underset{k=1}{\sum }}W_{o,k}^{m}*A_{k}^{m-1}+b_{o}^{m},$$ (14)

where:

• *k* is the number of channels in the image (1 for black and white and 3 for RGB)
• *o* indicates the number of channels in the output image
• * is the convolution product
• $A_{k}^{m-1}$ is the image input
• $W_{o,k}^{m}$ is the filter with which the convolution is performed
• $b_{o}^{m}$ is an additional bias

Once the feature maps are generated from the convolutional layer, an activation function is used to transform the values of the maps. A lot of activation functions exist, such as ReLU (Agarap, 2018). These functions are generally non-linear to adapt to a wider variety of data since a combination of linear functions is a linear function itself.

Pooling layers are used to downsample feature maps in order to reduce the computational load created by the convolutional layers. Pooling is most often performed as max pooling or average pooling. The pooling layer uses a filter of specified size (N x N) that slides over the whole feature map with a given step. Max pooling selects the highest value of the filter. Average pooling takes the average value of the filter. Their differences are given in the study by Zafar et al. (2022).Loss functions are primordial in all neural networks as they yield the error between the prediction and the truth. During training, this function is minimized. Many loss functions exist, and some are better suited for regression such as the mean squared error, while some are used for classification such as the categorical cross-entropy (Zhang and Sabuncu, 2018). To minimize these functions and obtain the best predictions, the stochastic gradient descent is used.

A very common problem in ML is overfitting. Overfitting is when a model works very well for the training data but performs poorly when fed new data. The most frequent reasons are as follows:

• The trained data is not fully representative of the potential input data
• The network is too complex for the problem at hand
• The network has been trained for too many iterations
• Data leakage

Hence, special attention was paid to overfitting in the proposed model. To make sure that the model was less likely to occur, dropout was implemented. Dropout deactivates a neuron with a given probability (chosen by the user) to reduce reliance on certain connections. As shown by Srivastava et al. (2014), this is an effective way to reduce the likelihood of overfitting. Another method to limit overfitting is by using L1 and/or L2 regularization to the weights (Ying, 2019). L1 and L2 both have their strengths and weaknesses, as explained in the study by Ng (2004). Hence, a combination of both called the elastic net, presented in Zou and Hastie (2005), further reduces overfitting. The new loss function becomes:

$$\begin{array}{c} E_{\mathrm{NET}}\left(\theta \right)=J\left(\theta \right)+\lambda_{L1}\overset{p_{L1}}{\underset{j_{1}=1}{\sum }}|\beta_{L1,j_{1}}|+\lambda_{L2}\overset{p_{L2}}{\underset{j_{2}=1}{\sum }}\beta_{L2,j_{2}}^{2}, \end{array}$$ (15)

where

• *J*(*θ*) is the existing cost function
• *θ* is the parameter to minimize
• *j* is the sum index (L1 or L2)
• *λ* is the regularization factor (L1 or L2)
• *β*_*j*_ is the weighting penalty (L1 or L2)
• *p* is the chosen degree of regularization (L1 or L2)
• *E*_*NET*_(*θ*) is the new cost function with L1 and L2 regularization

At last, comparing the loss function values of the training and validation dataset is a good metric to check overfitting.

#### 2.4.2 Proposed architecture

To construct the CNN for multipath mitigation, TensorFlow (Abadi et al., 2016) and Keras (Chollet, 2015) were used as the backbone. TensorFlow is an open-source deep learning library owned by Google that enables one to develop a machine learning algorithm from end-to-end. Keras is also an open-source machine learning library that runs on top of TensorFlow and allows one to easily build networks and train models. The images are grey-level to reduce the number of parameters to be trained, with a 256 × 256 pixel size. As there are, at most, 101 correlators used, the images were upscaled on the correlator delay axis. On the temporal axis, the images were slightly upscaled for E1-B (250 values per correlator position per second), while they were downscaled for GPS L1 C/A (1,000 values per correlator position per second). The images were then passed to the CNN model described as follows. This model is a simple one; it was observed that a complex CNN was not needed to successfully detect multipath from the correlator images. Indeed, with respect to Munin et al. (2020), who also use CNN for multipath detection, the presented network uses one less convolutional layer, making it more lightweight. Furthermore, special attention was given to overfitting by implementing dropout and L1 and L2 regularization, while Munin et al. (2020) and Quan et al. (2018) do not mention this threat. As multipath and the filter can introduce correlation between data inputs, overfitting can be even more prominent when using post-tracking outputs.

The first layer is a convolutional layer made of 20 filters of size 3 × 3 with a stride of 1, computed using (12). This layer uses the ReLU activation function. Then, this layer is followed by a max pooling layer of size 2 × 2 with a stride of 2 × 2. The outputs of the 20 filter maps are flattened (passed into 1D) to a layer of 1 neuron with a sigmoid activation function which yields the result for the image:

• 0 → Not multipath
• 1 → Multipath

**FIGURE 4:**
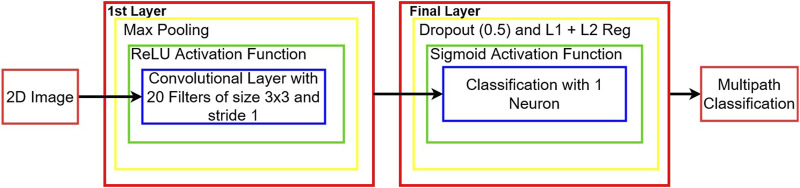
CNN architecture for multipath detection.

This layer is implemented with a dropout rate of 0.5, meaning that each neuron connection has a 50% chance of being ignored at each epoch. This layer is also regularized with the elastic net method given in (16) (L1 and L2 regularization with *λ* = 0.001 for both). The loss function chosen in this architecture is the binary cross-entropy. The batch size was of 64. The full architecture is given in Figure 4.

### 2.5 Dataset

The dataset was obtained from a dynamic scenario. The dynamic data were collected in all challenging conditions (suburban and deep urban). This was to obtain multipath errors to detect and exclude them. The dataset is on the L1 frequency band (1575.42 MHz) on GPS and Galileo with a 200 MHz sampling frequency. This large sampling frequency was used because it enabled a better reconstruction of the ACF over the span of the collect. An IFEN SX3 was used as the GNSS receiver (IFEN, 2023). The IFEN SX3 is a GNSS receiver that is capable of tracking all constellations and frequencies. It is very modular and can record raw data to then replay with different configurations. Indeed, the number and placement of correlators are selectable along with the loop filter bandwidths, EL spacing, etc.

This dataset was obtained with a car. Data collection took place in the city center of Toulouse, France, in a mix of urban and suburban environments for a total duration of approximately 40 min. The neural network had more than 4,000 images to be trained with, for each signal. There were more than 4 million correlators used per signal, and the satellite geometry was diverse. Having more multipath scenarios would likely be beneficial for the networks but the number of images and correlators was deemed sufficient to validate the proof of the concept. The assumptions were made on the material types because most buildings are made out of either concrete or bricks with glass windows, and the road, of asphalt. The IFEN SX3 was connected to a Novatel GPS 704-X pinwheel antenna. The data were replayed in post-processing to retrieve the full correlators for each epoch as it cannot be done in real-time with this number of correlators. The main parameters for each signal are given in Table 2.

**TABLE 2:** IFEN SX3 settings for GPS L1 C/A and Galileo E1-B.

	GPS L1 C/A	Galileo E1-B
Early–late spacing (chips)	0.125	0.04
DLL bandwidth (Hz)	1	1
Integration time (ms)	1	4
Number of correlators	101	101
Correlator sampling frequency (MHz)	200	200

## 3 Results

This section is divided into subsections: presenting the metrics used to analyze the results and the different configurations, the results for Galileo E1-B, and the ones for GPS L1 C/A. In order to investigate the benefits of using correlator images to detect multipath, the number of correlators used in images varies. To obtain accurate data labeling, the pseudorange bias has been computed with the most amount of correlators—101 at 200 MHz. In total, there are seven correlator variations presented in Table 3.

**TABLE 3:** Correlator configurations for GPS L1 C/A and Galileo E1-B.

Configuration name	Number of correlators	Correlator sampling frequency (MHz)
101@200	101	200
51@100	51	100
51@200	51	200
51@200&100	17 and 34	200 and 100
25@50	25	50
25@200	25	200
25@200&50	9 and 16	200 and 50

**FIGURE 5:**
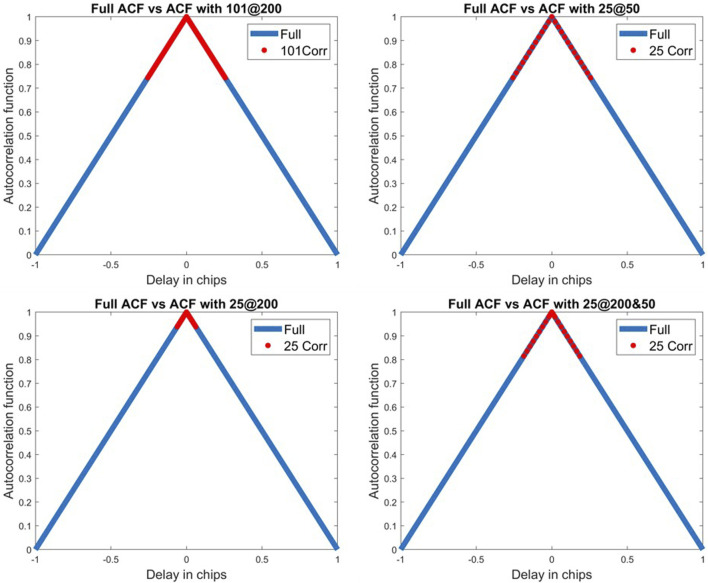
Illustration of some correlator variations for GPS L1 C/A.

Figure 5 illustrates the 101@200 correlators’ configuration along with the three configurations using 25 correlators by comparing the range of values obtained with the different variations *vs.* the full theoretical autocorrelation function for GPS L1 C/A.

**FIGURE 6:**
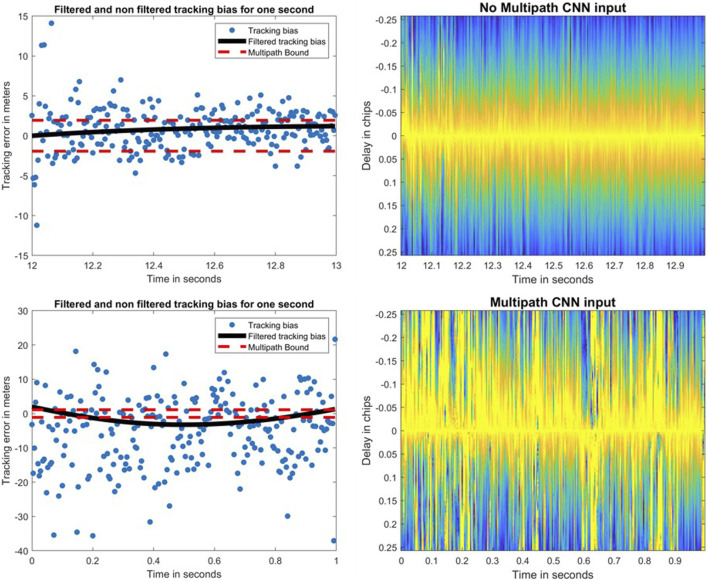
Filtered (black) and non-filtered (blue) tracking bias with its multipath bounds (red) for Galileo E1-B on PRN 3 (left) and their corresponding CNN inputs (right).

The results for these correlators will be presented for both signals. The images that are input to the CNN are the correlator values as a function of the chip delay (on the *y*-axis) with respect to the prompt correlator and time (on the *x*-axis) to yield 2D images. The CNN architecture and the labeled data remain identical no matter the correlator configuration. However, each correlator configuration induces a training period with the corresponding images of the chosen correlator configuration. Figure 6 represents the tracking bias and its filtered version for a given satellite on Galileo E1-B (left), while an example, from the training dataset, of both not multipath and multipath is given in Figure 6 (right) for the 101@200 correlator variation (colorized).

### 3.1 Result analysis

To train a binary classifier, the two classes—here, multipath and no multipath—need to be equally represented. Otherwise, this can lead to the network overpredicting the dominant class. This leads to a class imbalance in the datasets. There exist several countermeasures for this condition as given in the study by Buda et al. (2017). The chosen method is to downsample the dominant class. Indeed, the no multipath class will be reduced in size to roughly match the multipath one. To still train all images, the not-multipath images will be divided into several datasets and the final results presented in the following sections will be the average of all the trained models.

CNNs can also be overfit when the input data are too correlated. Hence, special care was taken to ensure that the input images were not correlated with one another. This correlation can come from the delay lock loop filter bandwidth. Indeed, the filtered pseudorange biases depend on the previous ones. To make sure that they were generally not correlated with one another, the cross-correlation factor [given in (16)] was computed between the filtered tracking bias outputs.

$$\rho_{x,y}=\frac{\mathrm{Cov}\left(x,y\right)}{\sqrt{\sigma_{x}^{2}\sigma_{y}^{2}}}.$$ (16)

For both signals, the cross-correlation factor was below 0.2, where most applications can be assumed as uncorrelated, after 1 s. The correlation of multipath was assumed to be less than the 1 s correlation caused by the DLL filter. Thus, the images were generated every 1 s.

**FIGURE 7:**
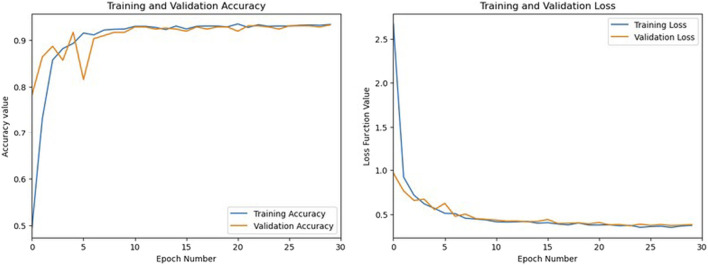
Accuracy and loss values over training iterations for training and validation datasets.

A good metric to check overfitting is the difference of the training loss with the validation loss of the binary cross-entropy function. The accuracy and loss values of the 101@200 configuration on Galileo E1-B are presented in Figure 7. As the difference in loss value is very small at the end of the training epochs (∼0.05), this shows the fact that the model is not affected by overfitting.

To analyze the results of a machine learning model, several metrics are often given: accuracy, precision, recall, and F score. Accuracy represents the proportion of correct classification the model made. Precision represents the proportion of correct multipath classification. Recall represents how well the model can detect multipath. The F score is the harmonic mean of precision and recall, and it better highlights the impact of false negatives and false positives than accuracy (Powers, 2020). The binary classification results can be represented as shown in the following table Table 4.

**TABLE 4:** Confusion matrix.

Truth ⧵ Actual	Positive	Negative
Positive	True Positive	False Negative
Negative	False Positive	True Negative

The accuracy, precision, recall, and F score are equal to (Dalianis, 2018):

$$\begin{array}{cc} A_{cc} & =\frac{TP+TN}{TP+TN+FP+FN}\\ P_{\mathrm{rec}} & =\frac{TP}{TP+FP}\\ R_{ec} & =\frac{TP}{TP+FN}\\ F_{\mathrm{score}} & =\frac{2P_{\mathrm{rec}}\times R_{ec}}{P_{\mathrm{rec}}+R_{ec}}. \end{array}$$ (17)

To better highlight the benefit of using CNNs for multipath prediction, these metrics will also be computed with the tracking bias method when the number of correlators is reduced. This means that the pseudorange bias will be estimated for each correlator configuration. Their accuracy, precision, recall, and F score for that configuration with respect to multipath classification performed with 101 correlators at 200 MHz will also be computed.

The difference between the time taken to predict the presence of multipath with the use of CNN with respect to the tracking bias method will also be presented. To have a fair comparison, both methods are compared when using the same number of correlators and are evaluated in the same coding language and on the same hardware.

### 3.2 Galileo E1-B

**FIGURE 8:**
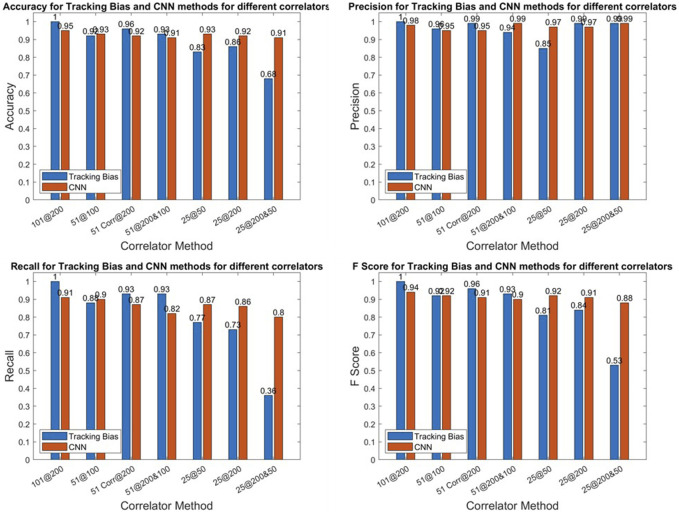
E1 metrics of the CNN.

In Figure 8, the accuracy, precision, recall, and F score are given for all correlator combinations for both CNN and tracking bias-based multipath classification. The tracking bias results are in blue, while the CNN results are in orange. The CNN results presented here are from the test dataset. The data split was 80% for the training data and 20% for the test data. As there is just one model and the hyperparameters of the model are not varied, no validation data were used to generate the results presented.

#### 3.2.1 CNN *vs.* tracking bias

The tracking bias method, when using 101@200, is the configuration used for determining the truth, so its metrics are all equal to one. When using 51 correlators—no matter the correlator sampling frequency—the tracking bias method performs better or at least equivalently to CNNs as conveyed by the higher F score and accuracy results. But when only 25 correlators are used, both F scores and accuracies plummet for the tracking bias approach. When the number of correlators is decreased, the tracking bias metrics quickly degrade in performance. This is shown by 11, 7.5, and 36% F score differences between 25@50, 25@200, and 25@200&50 configurations, respectively.

To compute the tracking bias, the reconstructed discriminator function linearly interpolates the delay when there is a zero crossing. By reducing the number of correlators and the correlator sampling frequency, the linear interpolation between two delays is greater and leads to a higher uncertainty on the resulting tracking bias based on the effect of multipath and noise at this time. On the other hand, reducing the number of correlators while maintaining a high correlator sampling frequency leads to a shortened discriminator function where the zero crossing may not be present. In that case, the tracking bias cannot be estimated. The metrics highlight that when the number of correlators is reduced, the CNN method outperforms the tracking bias one.

#### 3.2.2 Computational load

Table 5 shows the time taken by each method, normalized by the most time-consuming method. This table illustrates that all CNN-based configurations are two to six times faster than their tracking bias counterpart. This results from the tracking bias being computed 250 times per second since the integration period used here was of 4 milliseconds. On the other hand, since images are generated every second to limit cross-correlation, the CNN only predicts once per second, explaining the gain in time.

**TABLE 5:** Normalized mean execution time for all configurations.

	101@200	51@100	51@200	51@200&100	25@50	25@200	25@200&50
CNN	0.17	0.18	0.17	0.18	0.16	0.16	0.16
TB	1	0.51	0.80	0.70	0.32	0.67	0.41

#### 3.2.3 CNN correlator configurations

The 101@200 variation is the benchmark configuration for the CNN approach, but the overall performance of other correlator configurations, when looking at the accuracy and the F score, is very similar. This indicates that even with fewer correlators, the CNN used to detect multipath is still effective while using less computational power. This is seen when the correlator sampling frequency is lowered from 200 to 50 MHz, and the number of correlators is reduced by a factor of 4 for only a 2.8% reduction on the accuracy and 3% reduction on the F score. Thus, after this model is trained, the sampling frequency and the number of correlators could be reduced to alleviate some of the computational load while keeping a high correct classification rate.Despite the very high precision of the CNN from both correlator configurations of 51@200&100 and 25@200&50, their recall is lower with respect to other variations. It illustrates that the two configurations are the worst at detecting multipath. Even though this model is more precision oriented, the recall should not be fully sacrificed at the expense of precision. This is also reflected in the F scores of these variations as they have the worst F scores.The two CNN variations of 25@200 and 51@200 perform worse than 25@50 and 51@100 by 0.8 and 2% on their F scores, respectively, while outperforming the two configurations using 51@200&100 and 25@200&50. Thus, the multipath effect is more observable on a larger delay range of correlator outputs.

### 3.3 GPS L1 C/A

**FIGURE 9:**
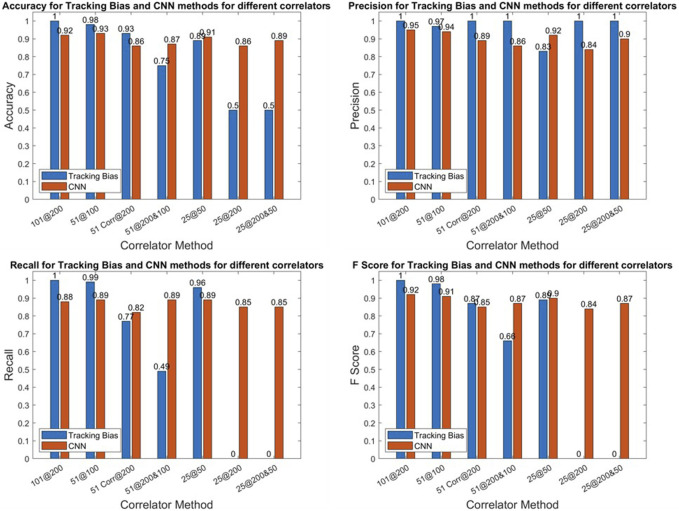
L1 metrics of the CNN.

In Figure 9, the accuracy, precision, recall, and F score are given for all correlator combinations for both CNN- and tracking bias-based multipath classification. The tracking bias results are in blue, while the CNN results are in orange.

The data split used here was the same as that for Galileo E1-B: 80% for the training data, 20% for the test data, and no validation data were used.

#### 3.3.1 CNN *vs.* tracking bias

For Galileo E1-B, the tracking bias method using 101 correlators is used for determining the truth explaining the perfect metrics. When using 51@100, the tracking bias configuration performs better because the range of the delay of the ACF is kept while maintaining a narrow enough linear interpolation. For 51@200, the F Score is 1.8% higher than that of its CNN counterpart. Despite having perfect precision, this variation has a much lower recall (77%), indicating that it is much worse at detecting multipath. This is further highlighted by the tracking bias results using 51@200&100. Indeed, the recall for this configuration is of 49.1% but has a perfect precision score.

When decreasing the number of correlators to 25, this becomes even more apparent as the 25@200 or 25@200&50 have recall scores of 0, meaning that these configurations classify every measurement as not affected by multipath. This can be explained by the fact that the early–late spacing is higher for GPS L1 C/A. Indeed, when the early–late spacing is increased, the linear region of the discriminator function is also increased. On the downside, the number of correlators needed to cover the linear region to find the zero crossing of the discriminator function increases. The zero crossing can even be shifted further in the delay domain due to multipath and noise. Hence, the two variations using 25@200 and 25@200&50 do not cover enough of the ACF to get a correct estimate of the tracking bias.

Moreover, when using 25@50, the F score of the CNN-based approach is 1.6% higher, which is due to its much higher precision with respect to the tracking-based approach despite its lower recall. Similar to Galileo E1-B, this conveys the fact that when the number of correlators is reduced, the CNN is a more viable solution to predict the presence of multipath than the tracking bias method.

#### 3.3.2 Computational load

Similar to Galileo E1-B, CNNs are much faster than the tracking bias method. Indeed, the CNN is 21 times faster on the 25@50 configuration as shown in Table 6. It is even more pronounced on GPS L1 C/A as the tracking bias is computed 1,000 times per second. The time taken by the tracking bias method also depends on the number of correlators used, whereas the CNN method’s execution time is almost identical. This can be explained by the fact that the CNN architecture is the same regardless of the correlator configuration. Therefore, the only change between correlator variations is the values of the filters and weights of the neurons. Since the time taken to generate an image is very low, this does not make a computational difference between the correlator inputs to the CNN.

**TABLE 6:** Mean execution time for all configurations.

	101@200	51@100	51@200	51@200&100	25@50	25@200	25@200&50
CNN	0.0085	0.0090	0.0091	0.0092	0.0093	0.0093	0.0093
TB	1	0.40	0.29	0.27	0.20	0.047	0.073

#### 3.3.3 CNN correlator configurations

From all the correlator variations, the CNN configurations using less correlators at a reduced correlator sampling frequency (51@100 and 25@50) have only slightly lower metrics than the CNN benchmark variation of 101@200 with respect to other correlator configurations. This can be explained by the higher early–late spacing chosen for this signal with respect to E1-B. Hence, the distortion caused by the multipath on the tracking bias is larger for higher EL spacing (Van Dierendonck et al., 1992). So, maintaining the same delay range on the ACF is more important with larger early–late spacing than having narrow correlators. Indeed, the decrease in the F score from 101@200 to 51@100 is of 0.33% and 1.14% from 101@200 to 25@50.

Furthermore, the two CNN configurations using 51@200 and 25@200 perform the worst. This is due to the fact that both of these variations do not show enough of the ACF—especially the 25 correlators’ configurations that show just enough correlators between the early and late correlators. Hence, these variations combine too little information on the ACF while still overcrowding the image with narrow correlators that do not add enough benefit to the classification.

For the CNN-based approach, the 51@200&100 to 25@200&50 variations show a performance that is in between the two previous configurations. As this configuration still requires a high sampling frequency of the signal and yet does not improve the classification, a lower correlator sampling frequency for correlators is favored for both computational and metric performances.

## 4 Discussion

In this paper, a theoretical classification of multipath has been proposed that uses GNSS tracking correlator outputs. This classification has been used to label data to train a convolutional neural network. Then, this CNN has been used to predict whether multipath was present or not on the correlator outputs from GPS L1 C/A and Galileo E1-B. This CNN has been developed to be low in complexity so that it can be trained with a low amount of data while avoiding under-- or overfitting.

The model uses tracking correlator values as a function of delay (with respect to the prompt correlator) and time in the form of images of 256 × 256 pixels. The inputs are lightweight, helping the predictions yielded by the CNN to be swiftly computed. This is illustrated by the fact that it is around 21 times faster to compute than the tracking bias for GPS L1 C/A and two to six times faster for Galileo E1-B, when using 25@50. The metrics between the CNN approach and the tracking-based approach were also compared, and the CNN performed better as the number of correlators was lowered. For example, when using 25@50, the F score was 91.8% for the CNN and 80.8% for the tracking bias on Galileo E1-B. The same conclusion applies to GPS L1 C/A as the F score was 88.9% for the tracking bias and 90.5% for the CNN with 25@50.

Several variations have been tested in the CNN to test how the CNN performs when reducing the number of correlators to generate the input images. The favored configuration is to decrease the number of correlators along with the correlator sampling frequency. Indeed, it was shown that by reducing the number of correlators from 101 to 25 and by reducing the resolution by a factor of 4, the F score only decreased from 94.7 to 91.8% on Galileo E1-B and from 91.6 to 90.5% on GPS L1 C/A. This highlights the robustness of this method to detect multipath and then exclude the corrupted measurement from the positioning solution.

Indeed, as mentioned previously, recall is how well the model detects multipath, thus excluding it. So, if a model correctly detects multipath, the confidence in the positioning solution would be higher. On the other hand, solutions that satisfy a certain precision requirement would improve the solution availability, i.e., having enough GNSS observations to have a full-rank system that can yield a GNSS position, velocity, and time (PVT) solution. As precision is how well the model classifies multipath, if there are very few false positives, then the model rarely falsely excludes useful measurements. In an accurate model, precision and recall are inversely correlated (Davis and Goadrich, 2006), meaning that increasing one would decrease the other. To increase the recall of the model, the threshold of the sigmoid function of the last neuron could be changed. The proposed decision in this study is as follows:

$$y=sigmoid\left(x\right),$$

$$\begin{array}{c} \left\{\begin{array}{c} y\geq 0.5\to \text{Multipath}\;\\ y<0.5\to \text{No Multipath}.\; \end{array}\right. \end{array}$$ (18)

By lowering the threshold to below 0.5, it would increase the recall at the expense of precision: it would only classify not multipath for very low values of *y*. In this paper, availability was deemed more important than integrity as shown by the models having higher precision than recall. This is because additional integrity algorithms can be added on top of this solution such as fault detection and exclusion (Rakipi et al., 2015). Orienting the model toward excluding all multipath could lead to fewer satellites than needed for a position computation. Furthermore, a limitation of the proposed method is that multipath-affected satellites on the brink of being labeled as multipath or not can degrade the positioning solution by being removed. Hence, the solution to mitigate this limitation is to improve the availability, and so the precision.

In the future, the number of correlators could be further reduced and their positions varied to investigate what could be the minimum number of correlators to classify multipath at a given rate. This could lead the method to be more implementable on real-time receivers so that the number of computed correlators is more achievable. Even 3D images can be built by taking into account the Doppler offset of the received signal, as done by Munin et al. (2020). Furthermore, other factors could be added for threshold determination such as the elevation of the satellite.

Excluding measurements because they are affected by multipath does not always lead to better positioning solution as it can lead to too few measurements to compute a position solution, or sometimes, this satellite was more important to the overall geometry than excluding the error was. Hence, the next step is to find a criterion to know if removing the multipath- affected measurement is worth it. Then, based on the results of the positioning accuracy, the multipath threshold can be redefined and optimized accordingly.

## Data Availability

The raw data supporting the conclusion of this article will be made available by the authors, without undue reservation.
